# Entropy and Gravity Concepts as New Methodological Indexes to Investigate Technological Convergence: Patent Network-Based Approach

**DOI:** 10.1371/journal.pone.0098009

**Published:** 2014-06-10

**Authors:** Yongrae Cho, Minsung Kim

**Affiliations:** 1 Division of Vice President, Science and Technology Policy Institute (STEPI), Seoul, Republic of Korea; 2 Graduate School of Technology and Innovation Management, Hanyang University, Seoul, Republic of Korea; 3 Graduate School of Innovation and Technology Management, Korea Advanced Institute of Science and Technology (KAIST), Daejeon, Republic of Korea; Katholieke Universiteit Leuven, Belgium

## Abstract

The volatility and uncertainty in the process of technological developments are growing faster than ever due to rapid technological innovations. Such phenomena result in integration among disparate technology fields. At this point, it is a critical research issue to understand the different roles and the propensity of each element technology for technological convergence. In particular, the network-based approach provides a holistic view in terms of technological linkage structures. Furthermore, the development of new indicators based on network visualization can reveal the dynamic patterns among disparate technologies in the process of technological convergence and provide insights for future technological developments. This research attempts to analyze and discover the patterns of the international patent classification codes of the United States Patent and Trademark Office's patent data in printed electronics, which is a representative technology in the technological convergence process. To this end, we apply the physical idea as a new methodological approach to interpret technological convergence. More specifically, the concepts of entropy and gravity are applied to measure the activities among patent citations and the binding forces among heterogeneous technologies during technological convergence. By applying the entropy and gravity indexes, we could distinguish the characteristic role of each technology in printed electronics. At the technological convergence stage, each technology exhibits idiosyncratic dynamics which tend to decrease technological differences and heterogeneity. Furthermore, through nonlinear regression analysis, we have found the decreasing patterns of disparity over a given total period in the evolution of technological convergence. This research has discovered the specific role of each element technology field and has consequently identified the co-evolutionary patterns of technological convergence. These new findings on the evolutionary patterns of technological convergence provide some implications for engineering and technology foresight research, as well as for corporate strategy and technology policy.

## Introduction

In recent innovation trends where technological developments take place rapidly, firms face the challenge of having to constantly develop new products through unceasing innovations. Thus, the complexity and diversity of technology have increased, resulting in radical technological innovations with drastic speed and intensity. Under this circumstance, ‘technological convergence’ occurs when there is a certain process of fusion among two or more disparate technology fields. Technological convergence is defined as the phenomenon where two or more existing element technologies with different functions combine to result in entirely new functions, which the existing technologies did not possess previously [1], [2].

One characteristic of convergence is that sometimes the technological revolution takes place through integration and recombination of the underlying knowledge convergence of other existing technology fields, rather than through developing a new technology [3], [4]. Additionally, each technology field and element technology takes a different role such as core function, integration, or commercialization. Convergence has been established as a prevalent concept, which explains the propensity and characteristics of current technological development. Considering these unique characteristics, many scholars have endeavored to identify the mechanism of the convergence phenomenon. Particularly, recent studies have paid attention to the increasing number of patents and interpreted the technological dynamics evident from the patent analyses [5], [6].

Despite the increasing interest in interdisciplinary technology, full-scale and extended studies to investigate dynamic patterns among heterogeneous technologies in the process of technological convergence have not been conducted and have rather remained stagnant. This situation is broadly due to the following reasons. First, technological convergence is fundamentally a complex process that blurs the boundary between industries and technologies [7]. In this regard, it is difficult to observe the pattern of advancement and development across disparate technologies based on a holistic view. Second, except for a few research studies, the lack of appropriate data on interdisciplinary research has been another impediment in measuring the convergence phenomenon among disparate technologies [8], [9]. Such problems have also led to the weakening of research on the phenomenon of technological convergence itself.

Accordingly, despite many researchers' efforts to identify the structure of technological convergence, most of them merely utilize the count information in journal publications or patents [10]–[12]. Such studies fail to investigate the dynamics of technological convergence. Furthermore, the broad definition of technological convergence at the product level and sometimes at the technology level has caused much confusion. These two levels of technology and product and the related research scope should be considered from separate viewpoints. On the one hand, at the product or industry level, technological convergence is commonly analyzed from the perspective of the merging phenomenon among disparate product components or functions or the related knowledge during the convergence [3], [4], [13]–[17]. Further, conceptual approach based on bibliographic information or more analytical approach to monitor the phenomenon of technological convergence from the industrial perspective has been attempted [2], [13], [14].

On the other hand, at the technology level, technological convergence is analyzed from the perspective of the roles and dynamics of each technology field during the convergence. However, existing studies on technological convergence were mostly related to the interdisciplinary or emerging technologies [18], [19]. Thus, even though they investigated the convergence phenomenon and provided good insights into technological developments, the study to reveal the dynamics of technology fields during convergence remains unexplored. This problem is more directly related to the issue of developing indicators to investigate the dynamic patterns of technologies.

In this context, the important research issues are to identify what kinds of structural patterns exist among the element technologies involved in technological convergence and which characteristics and roles such element technologies possess, as well as to show their changing propensities. We intend to pay more attention to the research on the roles and changes of each technology field in terms of the patent citation network and other relevant measures, rather than monitoring the convergence phenomenon in the conceptual or notional point of view. More specifically, our study focuses on the dynamics among the technology fields at the technology level. To do so, we investigate the dynamics of the roles, interactions, and changing propensities among disparate technology fields in the case of printed electronics, based on the postulation that this technology is the exemplar of technological convergence [20]. Against this background, we intend to answer the following research questions: “*How is each technology field involved and how does it maintain interactions with disparate technology fields during convergence?*” “*Which technology plays a central role in influencing other technology fields during convergence?*”

To overcome the drawbacks in the existing research, this study uses new quantitative indexes to which the printed electronics technology is subjected. By doing so, we can empirically measure the longitudinal patterns and characteristics of component technologies, consequently identifying the idiosyncratic pattern of interactions among disparate technology fields and their propensities during convergence. Printed electronics constitutes one of the representative convergent technologies. It is also well understood and explained with component technologies such as ‘*substrate*,’ ‘*ink*,’ ‘*circuit*,’ ‘*device*,’ and ‘*control*.’ These key component technologies comprising printed electronics can be clearly classified, enabling us to understand the main frame and basic structure of technological convergence. This study develops the entropy and gravity concepts as new explanatory indexes of the social phenomenon of technological convergence. In the existing studies, the entropy concept was used to investigate the characteristics of interdisciplinary research areas [21]–[23]. Meanwhile, the gravity model was used to reveal the trade flows in international economics [19]. Using the interdisciplinary approach, we address the social phenomenon issue of technological convergence with the concepts previously utilized in physics. More specifically, we attempt to develop and measure the degrees of citation activities among disparate technologies (entropy) and the degree of a certain technology's influence and force of attraction, compared with those of other technologies (gravity), according to each technology field by time periods.

To this end, this study calculates the entropy and binding force indicators from network visualization based on holistic perspectives. To do so, this study analyzes the citation relationships among the international patent classification (IPC) codes of the United States Patent and Trademark Office (USPTO). Therefore, we apply the entropy index to measure the degree of diversity and activities of patent citations among disparate element technologies such as *substrate*, *ink*, *circuit*, *device*, and *control*. On the other hand, we measure the binding forces among element technologies to assess certain technologies' influences on and forces of attraction toward others. Then we demonstrate entropy and binding force indexes using scatter plot graphs and perform nonlinear regression analysis employing these two indexes. By doing so, we can assess what kind of pattern is shown in terms of the activities and influences of each component technology field in the convergence technology, such as printed electronics. Furthermore, by observing the comprehensive measure of domination power among component technologies, we analyze the propensity of decreasing differences across heterogeneous technologies at each convergence stage.

In summary, understanding technologies in terms of the network-based approach is crucial to reveal the technological convergence phenomenon, since citation networks can provide a holistic view of the dynamics of complex relationships such as interactions and influences among heterogeneous technology fields. Furthermore, it is important to foresee promising technologies and their future propensities. Technological convergence possesses the intrinsic characteristics of complexity and diversity. In this context, a study using physical ideas provides new insights that can reveal the activities and forces of attraction in the technological convergence process.

### Printed electronics as convergence technology

Printed electronics is a groundbreaking technology used in manufacturing circuits and semiconductors. In contrast to previously existing technologies, it does not employ the photolithography method. Instead, printed electronics places inks with electronic characteristics that use printing devices for patterning. It results in manufacturing electronics products similar to producing printed publications such as paper products.

There are reasons for selecting the printed electronics technology to investigate the evolutionary characteristics of technological convergence. First, printed electronics combines printing technology (representative of old technology) with electronics technology (high technology). For this reason, we judge this as a symbolic convergence technology. Second, printed electronics is composed of element technologies that can be clearly differentiated. Third, with this technology's significance, its influence on society and industry is growing faster than ever. It can also be considered a disruptive technology, as it would largely replace the lithography process applied in electronic circuits, as well as the manufacture of various electronics products [24].

The printed electronics technology consists broadly of five component technologies: *substrate*, *circuit*, *ink*, *device*, and *control*. The *substrate* technology involves plastic boards, such as polyethylene terephthalate, polyethylene naphthalate, etc. The *circuit* technology entails the composition of circuits, considering the characteristics of printing methodologies. The *ink* technology refers to the manufacturing technology for inks with conducting, semiconducting, and insulating properties. The *device* technology relates to the printing machine and applicable components. Finally, the *control* technology adjusts the physical characteristics of each component technology in detail so that they can be integrated [25].

## Methods

In this study, we first collected patents related to printed electronics, as registered at the USPTO, to perform the analysis. To this end, we established search operators and the search period. Second, we extracted bibliographic information that encompassed the applicants of the patents by year and then assessed the citation relationships among the patents. Citation analysis is an efficient methodological approach for the measurement of activities and relevant interconnections among different parties or nodes [26], [27].

**Figure 1:**
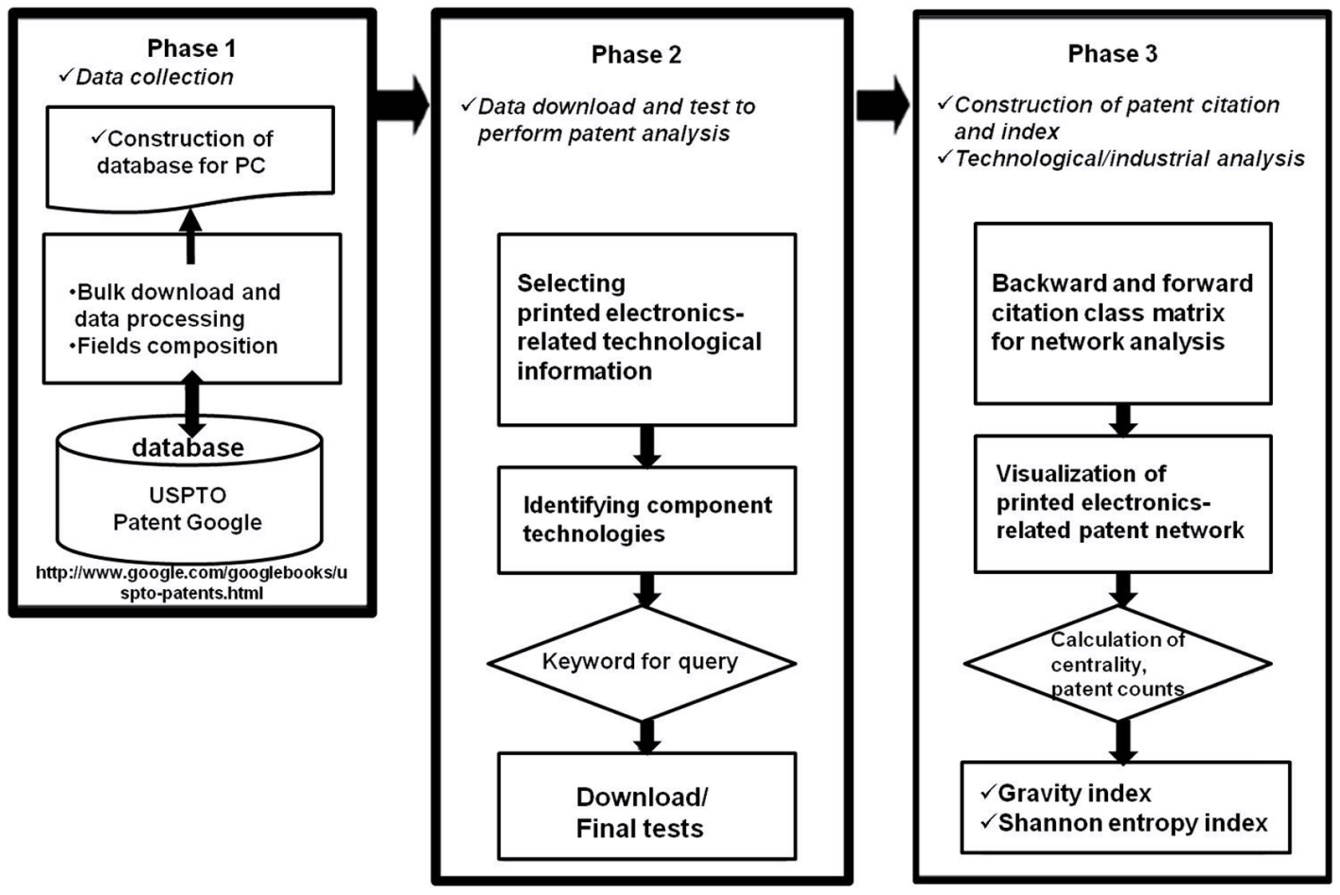
Research model for constructing USPTO patent database and patent citation network analysis.

In carrying out this study, we constructed a database on a PC using all the patent information that we downloaded, covering the registered and disclosed patents via the USPTO from 1976 to 2011. The data collection followed the process in Figure 1. The database has the following characteristics. First, since the full text of the USPTO is included, necessary fields for analysis indicators can be established. Accordingly, full-text information can be freely utilized, depending on the user's needs. The information that can satisfy such conditions is sourced from the full-text search at the USPTO's homepage. We downloaded the entire source codes via the webpage and utilized the data. Second, this bibliographic information is directly provided by the USPTO and is therefore not processed by database-specialized institutes or firms. Thus, we considered the possibility of omission of certain information. To prevent such potential limitations, we also utilized the Wintelips database provided by the Worldwide Intellectual Property Service, a Korean firm specializing in patent information management. Cross-checking between the USPTO and Wintelips databases can strengthen the validity and reliability of our database.

The main contents in Figure 1 are as follows. First, in Phase 1, the focus is on utilizing the USPTO database that includes patent citation information as the main source of information for patent analysis. Accordingly, the entire data for the USPTO patent is collected through the bulk download method, and data processing is carried out by employing the data mining method. Fields are created and a search database format is established using bibliographic information. Second, in Phase 2, the main keywords of the printed electronics technology are utilized over the patent database that records fields, including citation information, to collect bibliographic information.

Through this process, we collected patents related to printed electronics that were registered on the USPTO over the 1976–2011 periods. In detail, we collected data in the following order: establishing search operators and the search period, extracting patent bibliographic information over patents (including the given search terms during the period), and assessing the citation information across each patent. In this paper, patent bibliographic information, including combined words and individual words related to printed electronics in the abstracts and claims, was subjected to our analysis. Through the keyword extraction method, we identified 1,886 patents. As a result of extracting citation information by each patent based on such bibliographic information, we also obtained 75,443 counts of citation information. Based on the construction of bibliographic information and the corresponding citation datasets, we visualized patent networks and calculated network-specific indicators. Applying the indicators, we further calculated entropy and gravity indicators to investigate the activity and influence of each technology field. This process is reflected in Phase 3.

Subsequently, we broadly set the direction of analysis, utilizing the information in two ways. The first one was the reclassification by the characteristics of the technologies. To categorize the extracted patent information according to the components of the printed electronics technology, we referenced the IPC to which each patent belongs. The IPC has the section-class-subclass-main group-subgroup structure. The patents used in this study can be categorized under 51 types of IPC main groups. In this study, we classified the IPC main groups applicable to the five element technologies of printed electronics (Table 1), considering the definition of the main group (8th edition of the IPC), as well as the characteristics of the patents belonging to each group.

**Table 1:** Technology classification in IPC main group.

Technology	IPC main group
Device	B05C017, B21D053, B41C001, B41F005, B41F007, B41F013, B41F015, B41F031, B41F035, B41J002, B41J003, B41J023, B41J029, B41L013, B41M001, G01D018, G01N027, G03C001, G03C005, G03F007, G03G005, G03G009, G03G013, G03G015, H05B001, H05B003
Ink	C08F002, C08K003, C09D011, C09K011, H01B001
Substrate	B32B003, B32B009, B32B027, B32B031, B41M005
Circuit	H01K003, H01L029, H01R012, H05K001
Control	B05D001, B05D003, B05D005, B44C001, C25D001, C25D005, G01D015, H01L021, H01R009, H04N001, H05K003

The second one was the reclassification of patent information according to time periods. In conjunction with the classification of component technologies for printed electronics, we categorized the previously extracted citation information into four periods. Period 1 was set from 1976 to 1994, and from that point on, 5-year intervals designated 1976–1999 as Period 2, 1976–2004 as Period 3, and 1976–2011 as Period 4. To conduct our empirical research, we reconstructed the patent information and related citation information by each period and component technologies, according to the two analysis directions.

**Figure 2:**
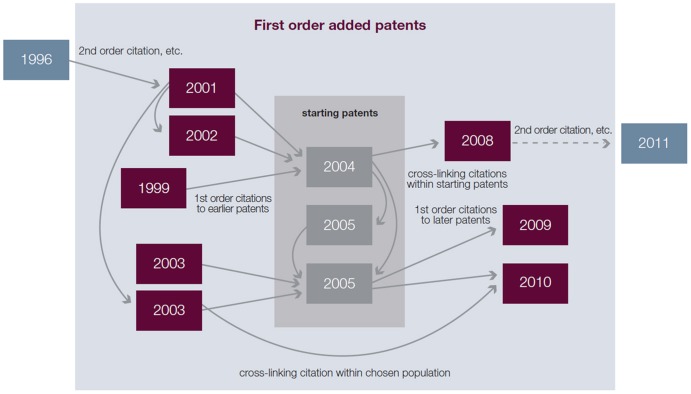
Structuring process of patent bibliographic information and citation information (reinterpreted and edited for reference [28]).

In terms of the extraction of the citation information from the bibliographic information, a processing structure can be visualized in a conceptual diagram, as shown in Figure 2 [28]. It illustrates a sample structure of citation steps and their relationships. More specifically, this illustration is provided as a sample to shed light on the structure and linkages among ‘starting patents,’ ‘first-order citations,’ and ‘second-order citations.’

If we apply the logic of this structure to the patent citations of our data, the patent information can be categorized into two types. The first group consists of the citation relationships among 1,886 starting patents. The second group consists of the citation relationships outside of the starting patents. In particular, the second group of patents comprises instances that are not applicable to the starting patents but cite or are cited by such starting patents. In this case, 14,460 instances have been additionally extracted. For structuring the patent network on which the citation information among patents must be based, we need the bibliographic information for the additional 14,460 patents (applicant's name, applicant's nationality, application year, registration year, etc.). In summary, regarding the bibliographic information, we utilized 16,346 counts of patent information totaling 1,886 patents that were actual analysis subjects and 14,460 patents that had citation relationships with these in establishing the citation network.

Furthermore, we eliminated the instances where overseas patents (not US patents) were included in either the cited or the citing patents. We also removed the instances where the bibliographic information omitted data on the citing or the cited patents. Finally, we eliminated the instances of citation information outside of the starting patents, because they were deemed unnecessary for the actual research analysis. In summary, 23,110 entries of citation information out of 2,689 entries of bibliographic information over the 1976–2011 periods were subjected to final analysis.

After conducting the aforementioned data processing, we developed the following two indexes based on physics in order to identify the phenomenon of technological convergence. We expect to make research contributions by providing grounds for the argument for the evolution of technological development based on scientific methodology.

### 1. Network visualization

Most scientometric/bibliographic data-based networks can be represented by graphs. This applies to co-authorship and collaboration networks as well as to cross- or co-citation networks [29]. Furthermore, network visualization and the related analysis generates new and valuable information, allowing better design and strategic planning, enabling decision makers to characterize network components by area of work, identify entities playing major roles as central hubs or located at critical network positions [30]. In this sense, network visualization and the relevant analysis in this study provide the linkage pattern and its evolutionary dynamics of interactions among different technology fields in the process of technological convergence.

**Figure 3:**
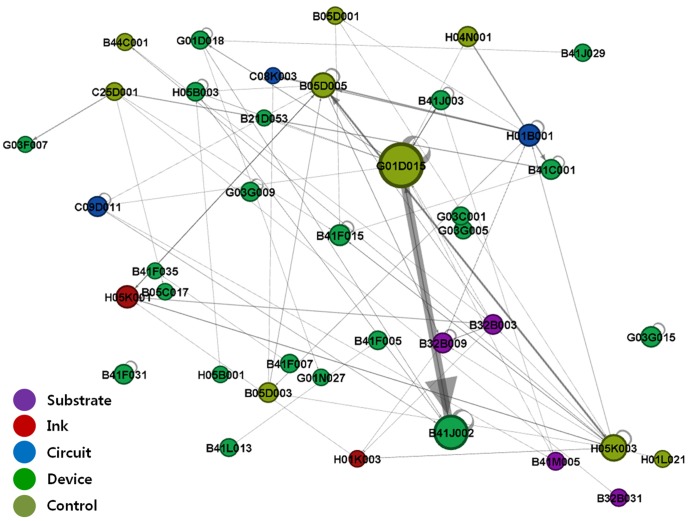
Network visualization by technology fields of IPC codes (1976–1994).

**Figure 4:**
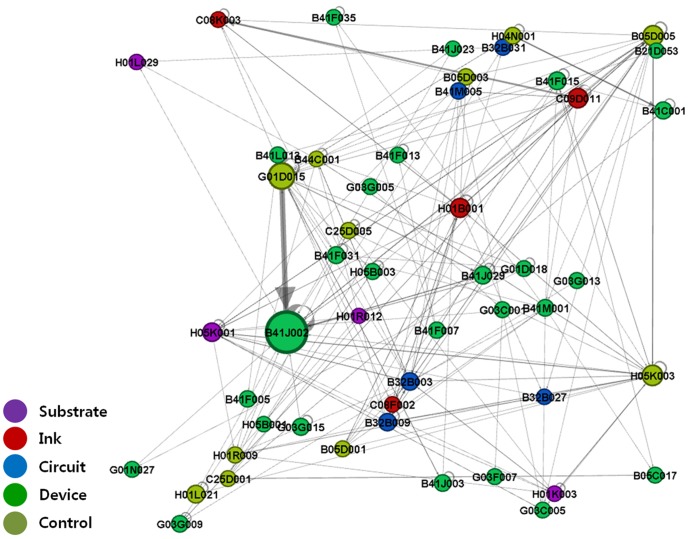
Network visualization by technology fields of IPC codes (1976–1999).

**Figure 5:**
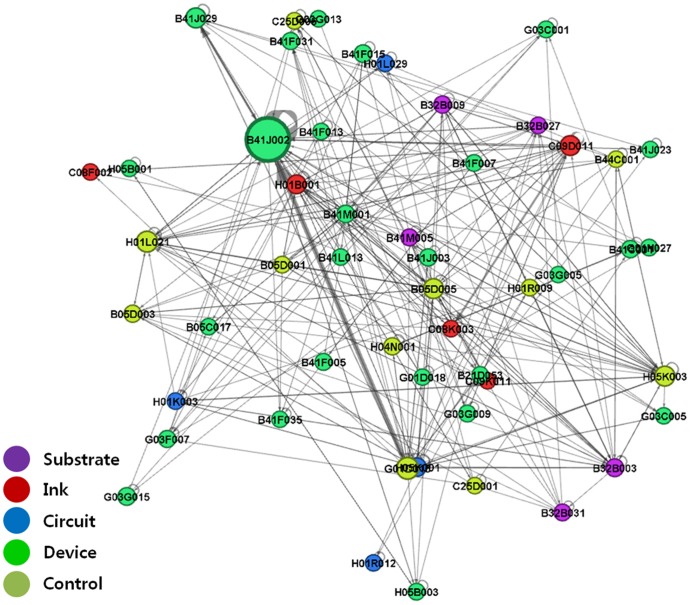
Network visualization by technology fields of IPC codes (1976–2004).

**Figure 6:**
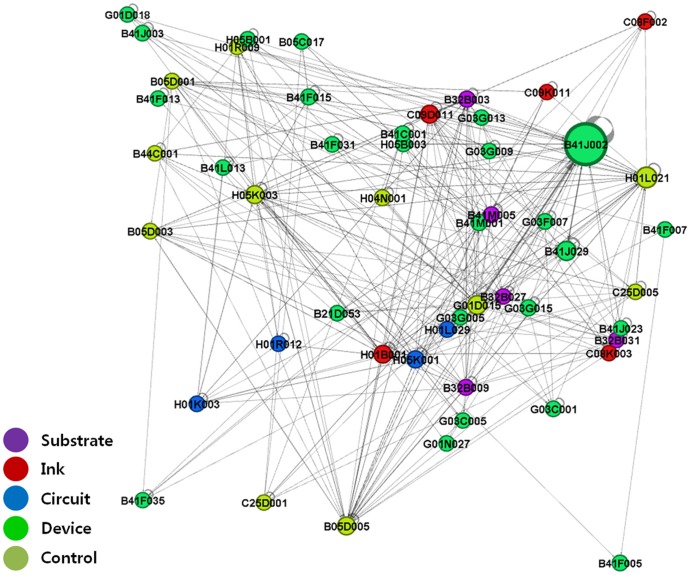
Network visualization by technology fields of IPC codes (1976–2011).

We performed IPC-level network analysis by substituting patent nodes with IPC code nodes to show the structure of the technology fields related to printed electronics. Based on the citation direction and with links shown as arcs, the IPC codes that cited other IPCs' patents were visualized as receiving inflows of technological knowledge, while those whose patents were cited by others were visualized as providing outflows of technological knowledge [31]. Figures 3–6 show IPC code-specific patent networks related to printed electronics technologies. The node size indicates the total number of patents that belongs to each IPC code.

Figure 3 shows active knowledge outflows and inflows among the IPC codes that owned *device* technologies and *control* technologies, being the main players in printed electronics during the 1976–1994 period. Overall, we found certain IPC codes (e.g., G01D015 and B41J002) that existed in the central position, while maintaining interactions with one another, and influenced other IPC codes. Figure 3 shows that G01D015 of the *control*-related IPC code occupied the center of the network, influencing *device*-related IPC codes and maintaining a strong outflow linkage to B41J002 of the *device*-related IPC code. In other words, the main technological knowledge generally flowed from the *control*-related technology to other types of technologies.

The patent citation network (Figures 4–6) exhibited more complex structures from the mid-1990s to the 2000s. Specifically, the technological linkage structures had become more complex, and various technology fields (nodes of IPC codes) occupied the center of the networks. Particularly, compared with the case in the first period (1976–1994), the *device*-related technology fields (e.g., B41J002) maintained the central position of the network structure, and their patent citation actively occurred with other technology fields. In other words, leading IPC codes that played a central role during technological convergence consistently influenced other IPC codes in terms of knowledge flow, regardless of network periods. Additionally, a strong link between B41J002 (*device*) and G01D015 (*control*) continued. In the case of the *ink*-related technology fields, more complex and active relationships with other technology fields were observed, compared with the situation in the first period. For example, focusing on the *ink*-related citation linkages showed that the links had become more scattered and complex than those in the first period. H01B001, C08F002, and C09D011 had increased their overall flows of technological knowledge with disparate technology fields. They had also diversified their citation linkages with other technology fields.

### 2. Entropy

For the investigation of technological development and evolution during technological convergence, citation information provides important clues by directly or indirectly indicating the interactions and connections among disparate technology fields. Additionally, not only the entire network, but also the individual networks of nodes among the other nodes can show idiosyncratic patterns of how the network of each node changes during technological convergence. In this study, we use the entropy and gravity concepts to analyze the relationship between a technology field and the others in the IPC-code level.

The entropy concept, expressed as the Shannon entropy, is used to analyze the relationship between the main technology and the others. With regard to the Shannon entropy concept, previous studies developed quantitative indicators to measure the interdisciplinary phenomenon in how many scientific/technological subfields and how intensively is engaged with diversity [21], [23]. Furthermore, we found additional research that investigated technological fusion in terms of technological foresight and changing patterns [18], [19]. In particular, for the evaluation of the scientific knowledge variety, researches used the information entropy of the shares of journal subject categories allocated by publications [19], [23].

These papers also utilized the Shannon entropy to measure the technological fusion or interdisciplinarity among disparate technology or industry fields. In this study, we analyze and investigate the roles of and characteristic interaction patterns among technology fields during technological convergence. To this end, we study the citation structure of each IPC code, among the other IPC codes, by applying the entropy concept. The Shannon entropy [32] is defined as follows:
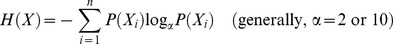
(1)where *P* (*x_i_*) is the rate of citation diversity, which is distinguished by the *n* node. Moreover, the number of state *i* is related to the heterogeneous citation activities in the individual network. Thus, *P* (*x_i_*) corresponds to the number of patents involved in a citation of technology field *i* divided by the total number of patents involved in the citations of all technology fields in the individual IPC code. In detail, if the citation activities exist in the same IPC code, the number of state *i* is the only one related to zero-interaction with the different technologies during technological convergence. In this case, the value of *P* (*x_i_*) is 1, and the value of Shannon entropy is 0. Therefore, the entropy concept is needed for analyzing the degree of citation activity of a technology field among the citations in which disparate technology fields are involved. If the diversity of a technology's activity is larger, the Shannon entropy value increases. In other words, the increase of the Shannon entropy value means the increase of citation activities with heterogeneous technologies.

### 3. Binding force

Gravity is commonly known as the force of attraction among objects. If we apply this concept as an attraction force in the network analysis, the gravity concept can be used to know how each node is cohesively connected with the others. In this sense, the gravity concept is directly related to the concept of the degree of each node's attraction, compared with those of other nodes. Conceptually, when the size of central node and *n* node which is connected to the central node becomes larger, the corresponding interaction and connection between them becomes larger accordingly. In this process, the binding force value of the central node increases and it attracts other nodes. This phenomenon also means that interaction and exchange are active among *n* nodes. Applying the gravity formula, we define the binding force as follows:
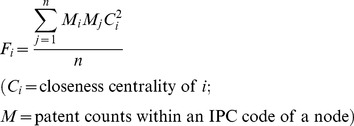
(2)


Consequently, equation (2) can be used to explain the binding force of node *i* among *n* nodes. Closeness centrality is an index describing how much node in a network is closely connected to the other node. Also, closeness centrality is conceptually defined as the inverse value of geodesic distance [33]–[35]. In this sense, closeness centrality can be a proxy variable to explain the gravity concept.

However, the description of closeness centrality is insufficient to fully describe the binding force of each node network. Therefore, we develop the closeness centrality for the binding force using the gravity concept. Note that the binding force is the force of attraction that acts among each other's particles through action and reaction in the physical space. From this concept, we calculate and explain the binding force of node *i* that is connected to the other node. In this study, closeness centrality, which is used for our analysis, is related to distance, because it also has geodesic quantity. Additionally, mass (*M*) is the size of a node, which is the number of patents possessed by an IPC code.

### 4. Nonlinear regression fitting

As an additional analytical method, we use nonlinear regression to explain the evolutionary patterns and efficiency of technological convergence in printed electronics. We suggest the disparity concept among technology fields as the representative analytical indicator in our study. Changes of disparity can show and explain the evolution and development of interactions among the component technologies involved in technological convergence. We also define and develop the ‘domination power’ as a comprehensive concept that combines the citation activity (Shannon entropy) and force of attraction (gravity) among the IPC codes of nodes. The changes of disparity of each node's domination power provide significant implications for the assessment of the degree of activity and attraction simultaneously in the process of technological convergence. In this study, we intend to find unique patterns of disparity in the patent citation network by using the nonlinear regression method. The equation of nonlinear regression in each period is as follows:
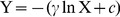
(3)where we define the axis of *Y* as the rate of the maximum value of the Shannon entropy and the binding force in a selected period, and the axis of *X* as the inverse value of patent counts in an IPC code, expressed as:
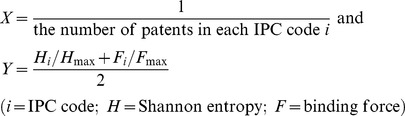
(4)where *Y* is the domination power of each IPC code in the whole network. As the technological convergence accelerates, the technological disparity of the individual IPC codes is deemed to have a decreasing pattern. The reason is that technological convergence exhibits the characteristics of raising the interactions among disparate technology fields, resulting in the decrease of heterogeneity gaps among technology fields. Conversely, we can speculate that the disparity among technology fields is high in the early phase of technological convergence. Thus, the γ value indicates the technological disparity. Through the total period, the technological disparity is decreased when the γ value is decreased.

### 5. Networking size

The magnitude of the networking size can be evaluated by comparing it with the Shannon entropy and binding force values in each period. The *X*-axis means the period, and the *Y*-axis indicates the networking size, defined as:
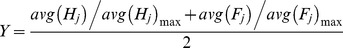
(5)where 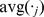 is the average value of *j* period, and 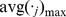 is the maximum average value in the total period. Equation (5) is used in a similar way as the equations (3) and (4) of domination power in subsection 3.4 to investigate and assess the efficiency during technological convergence in printed electronics.

## Results

**Figure 7:**
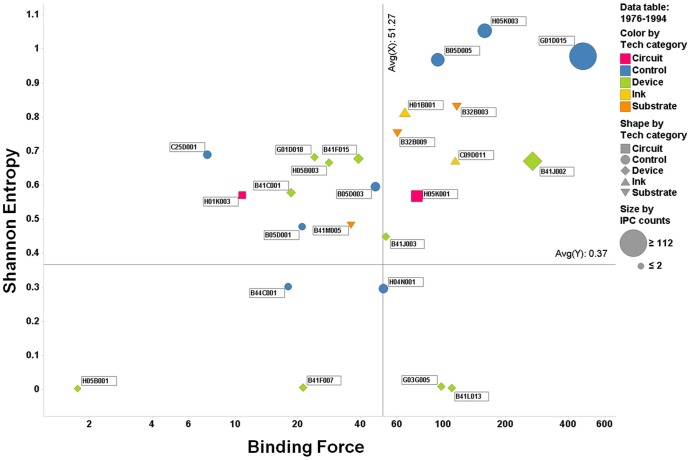
Scatter plot of IPC codes from 1976 to 1994 (first period).

**Figure 8:**
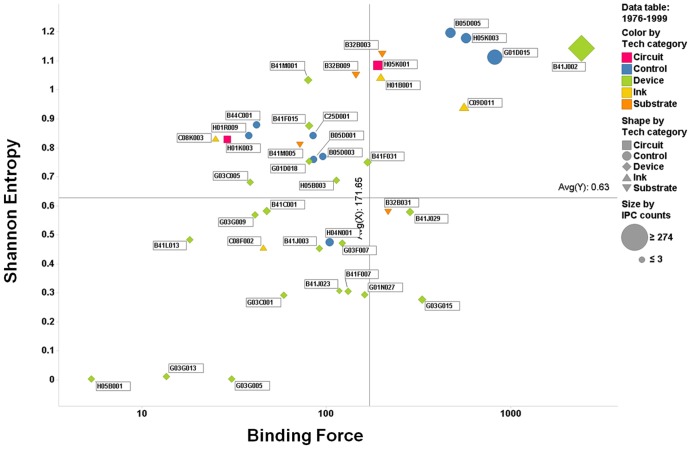
Scatter plot of IPC codes from 1976 to 1999 (second period).

**Figure 9:**
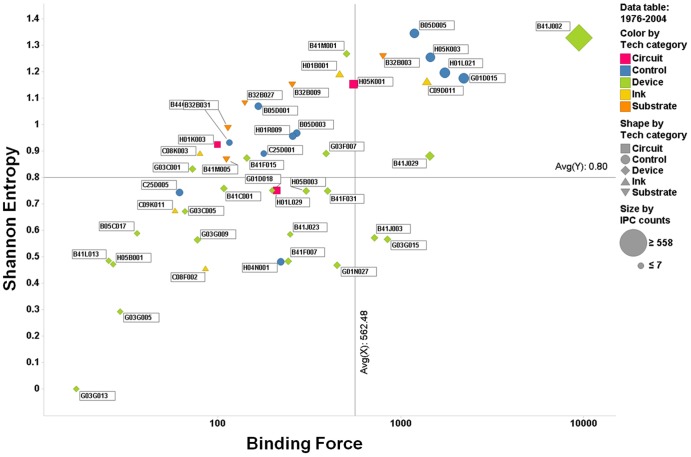
Scatter plot of IPC codes from 1976 to 2004 (third period).

**Figure 10:**
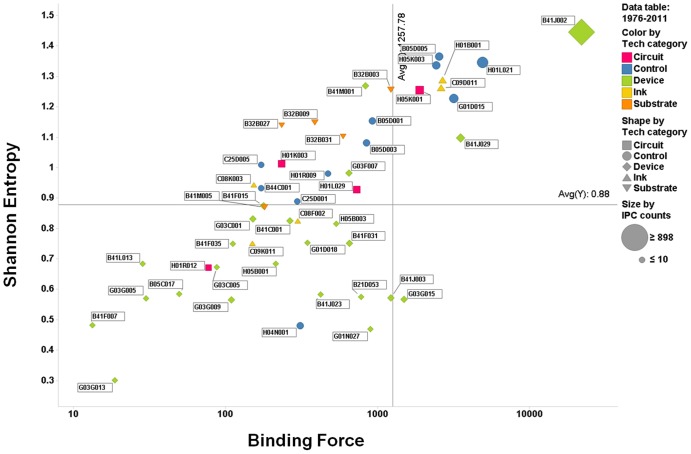
Scatter plot of IPC codes from 1976 to 2011 (fourth period).

To better understand the evolution of individual IPC codes within the patent network, we constructed a two-dimensional quadrant with the axis of the Shannon entropy and the binding force. Specifically, this matrix was depicted for the four periods, with the binding force indicated on the abscissa and the Shannon entropy on the ordinate (Figures 7–10). This matrix enables us to understand the dynamics of the interactions, represented as activity and force of attraction, and their changing patterns among heterogeneous technology fields of the IPC codes. The size of each circle (or other types of figures) in the matrix represents the total patent counts for each IPC code. Two lines on each positioning map can be used to create a scatter plot that indicates the average values of the indicators (vertical: binding force, horizontal: Shannon entropy).

The first quadrant is defined as having both high binding force and high Shannon entropy values. Thus, we can assume that the citation activity and the binding force are vigorous in the patent citation network, and this phenomenon is deemed as one of the typical results of technological convergence. The second quadrant is defined as the low binding force and high Shannon entropy quadrant. Technologies belonging to this quadrant tend to have active interactions among the technology fields in the convergence, but they have a lower binding force of technological leadership and attraction. This means that the technology field within this quadrant has the potential of technological convergence. In the third quadrant, both the binding force and Shannon entropy values are low, indicating that the technologies within this quadrant have not shown either active interactions with other technology fields or forces of attraction toward other technology fields during the convergence process. Finally, in the fourth quadrant, the binding force value is high, whereas the Shannon entropy value is low. The force of attraction of a technology field tends to be high, but the degree of the diversity of activity and frequency of citation is relatively low. Our analysis results using the gravity and Shannon entropy indexes are depicted in Figures 7–10.

### 1. 1976–1994 (first period)

In this period, various element technologies such as *substrate*, *circuit*, *ink*, *device*, and *control*, which were involved in printed electronics, started to emerge, and the *control*-related technologies occupied the highest position. Additionally, the IPC code of B41J002 (*device*) had high binding force and Shannon entropy values. We can assume that B41J002 connected with the other technologies, such as *substrate*, *circuit*, *ink*, and *control*, where the *device* technology played the role of integration with disparate technology fields in the printed electronics process. The IPC code of G01D015 (*control*) had the highest binding force and Shannon entropy values, positioned in the first quadrant; H05K003 and B05D005 were also in a similar position. When we consider the characteristics of the printed electronics manufacturing process, the IPC codes of G01D015, H05K003, and B05D005 of the *control* technology seemed to have reduced the gaps among the different characteristics of the technology fields in printed electronics. The technologies of the first period are randomly distributed in the quadrant. Therefore, the unique pattern of interactive relationship seemed to be weak in this period.

### 2. 1976–1999 (second period)

More element technologies of IPC codes entered and emerged in the second period. Visible changes were presented where the number of *device*-related technologies began to increase. As mentioned, these technologies played an important role in the integration among disparate technologies, eventually materializing the printed electronics process. Therefore, the emergence of various *device*-related technologies was vital in the development of the printed electronics technology. However, most of the Shannon entropy values of the *device*-related technologies in the period were relatively low, compared with those of other technology fields. Thus, there seemed to be few citation activities of the *device* technologies. Moreover, B41J002 had the highest binding force and Shannon entropy values out of the entire technology fields of IPC codes, showing a different trend from that of the first period.

### 3. 1976–2004 (third period)

In the third period, more diverse relationships were formed among the technology fields, and the forces of attraction among disparate technologies also increased in printed electronics. For this reason, technology fields showed a relatively small degree of dispersion, more concentrated on the average values of the binding force and the Shannon entropy. Moreover, these technologies gradually showed a proportional relationship between the binding force and the Shannon entropy. In this context, it is assumed that the binding force of technology can have a positive relationship with the Shannon entropy.

### 4. 1976–2011 (fourth period)

In this period, the *device* IPC codes reached the largest number in the network from the first to the fourth periods. The number of nodes associated with the *device* technology was the largest in the network, and most of the Shannon entropy values of the technologies associated with the *device* were distributed below the average values of the Shannon entropy and the binding force. However, the B41J002 code continued to be positioned in the highest region in the first quadrant and had the highest binding force and Shannon entropy values in the whole network. B41J002 is called the “inkjet printer and ink dryer technology.” From the industrial perspective, the B41J002 code can be considered as a main technology related to the materialization and embodiment of the printed electronics process, particularly involved in the nanoparticle ink technology. Therefore, we can assume that this technology field plays an essential and direct role in the completion of the technological convergence process.

Most *control* technologies are distributed within quadrants 1 and 2. The *control* technologies in quadrant 1 are related to the *ink*-related technologies. In the process of technological convergence, the *control*-related technologies in quadrant 1 enable the *ink*-related technologies to be converged functionally with the other technologies. Moreover, regarding the printed electronics industry, the *control* technologies of quadrant 2 help the *circuit*- and *substrate*-related technologies to be functionally converged. Throughout the periods, the *circuit*- and *substrate*-related technologies had not increased their Shannon entropy and binding force values, which means that they had played the role of platform providers with basic element technologies.

The Shannon entropy values in most of the *substrate*, *circuit*, *ink*, and *control* technology fields were higher than average. For this reason, the *circuit*-, *control*-, *ink*-, and *substrate*-related technologies seemed to have high degrees of interactions with one another in various ways for technological convergence. Additionally, most of these technologies also display high binding force values in quadrants 1 and 2. This result indicates that these technologies tend to have strong relationships in their patent citation networks. In other words, we found that these technologies in quadrants 1 and 2 contribute to make new products and develop new technologies.

In summary, a much stronger correlation between the Shannon entropy and the binding force was evident in the fourth period than that in any other period. In other words, the propensity of integration among disparate technology fields through technological interactions had been strengthened for the total period.

### 5. Nonlinear regression

**Figure 11:**
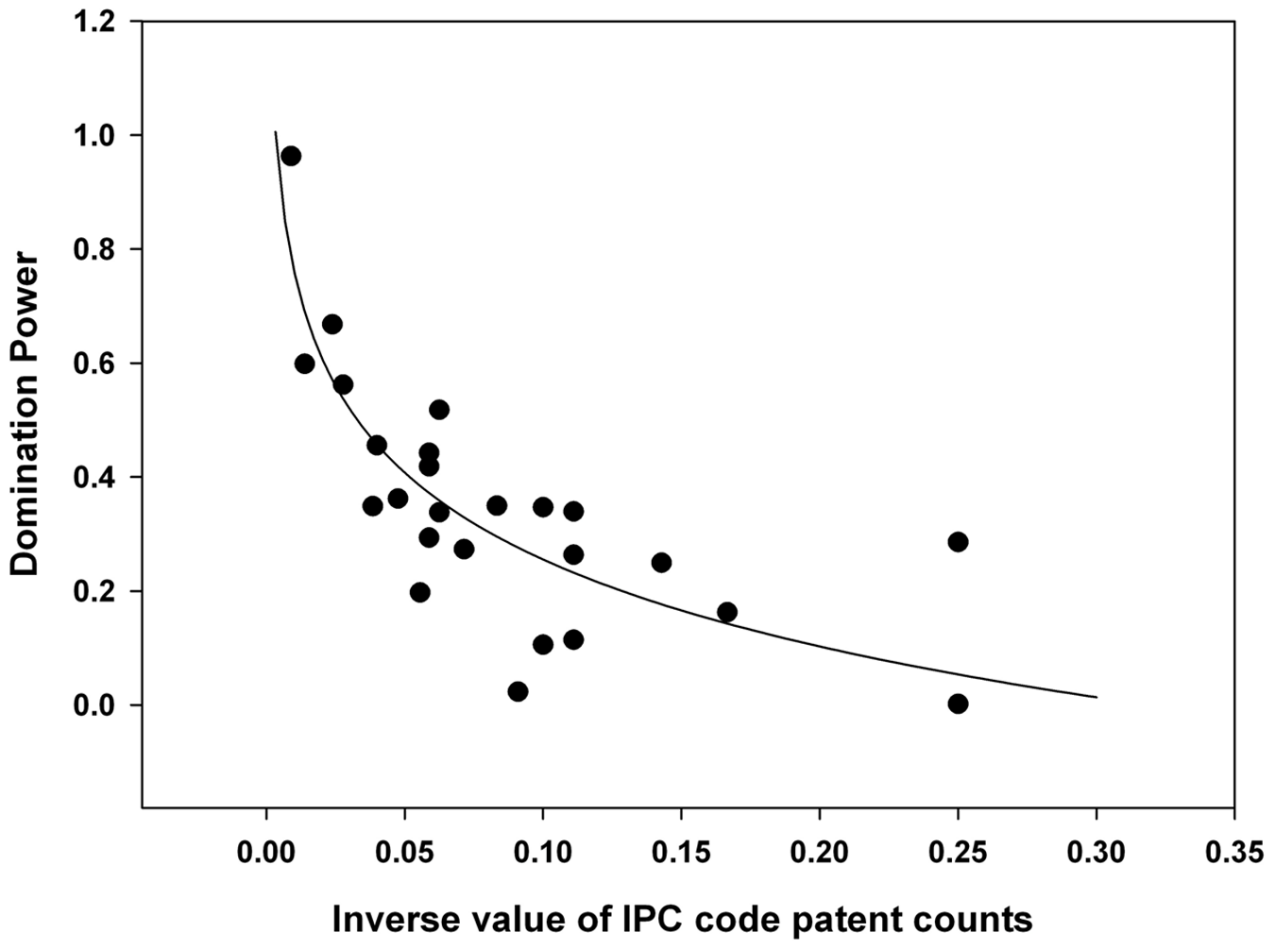
Nonlinear regression from 1976 to 1994 (first period), γ = 0.2200.

**Figure 12:**
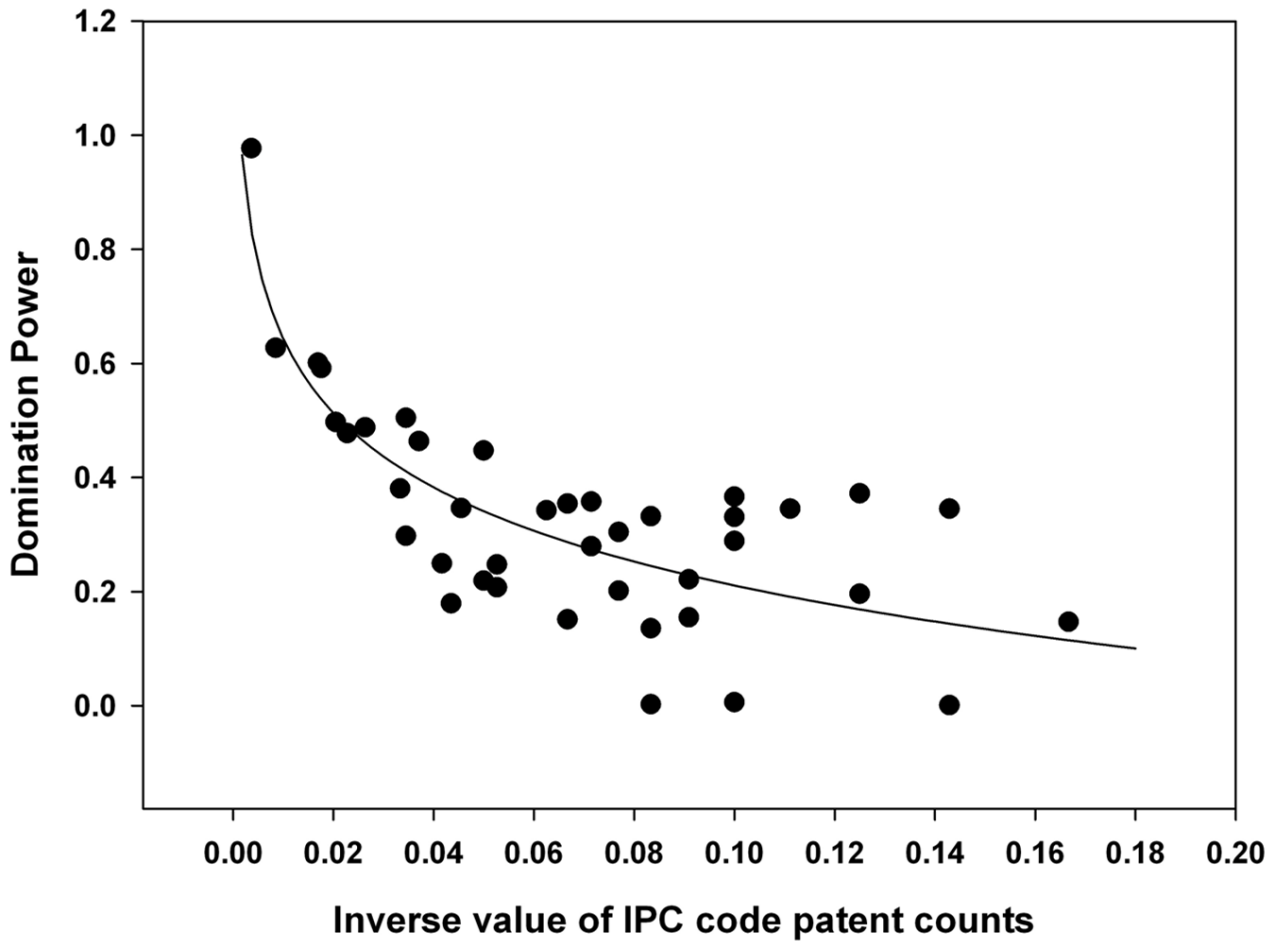
Nonlinear regression from 1976 to 1999 (second period), γ = 0.1878.

**Figure 13:**
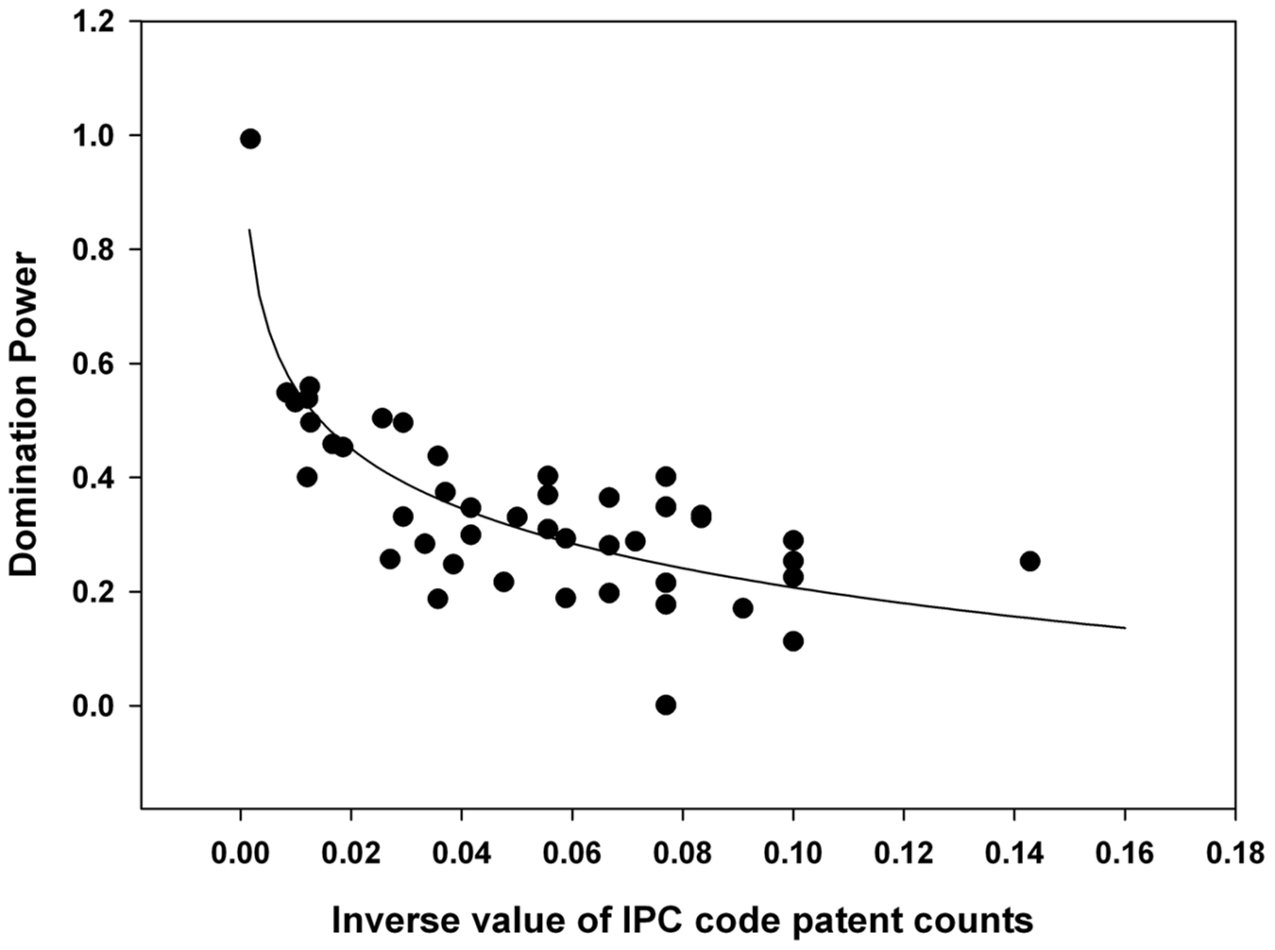
Nonlinear regression from 1976 to 2004 (third period), γ = 0.1511.

**Figure 14:**
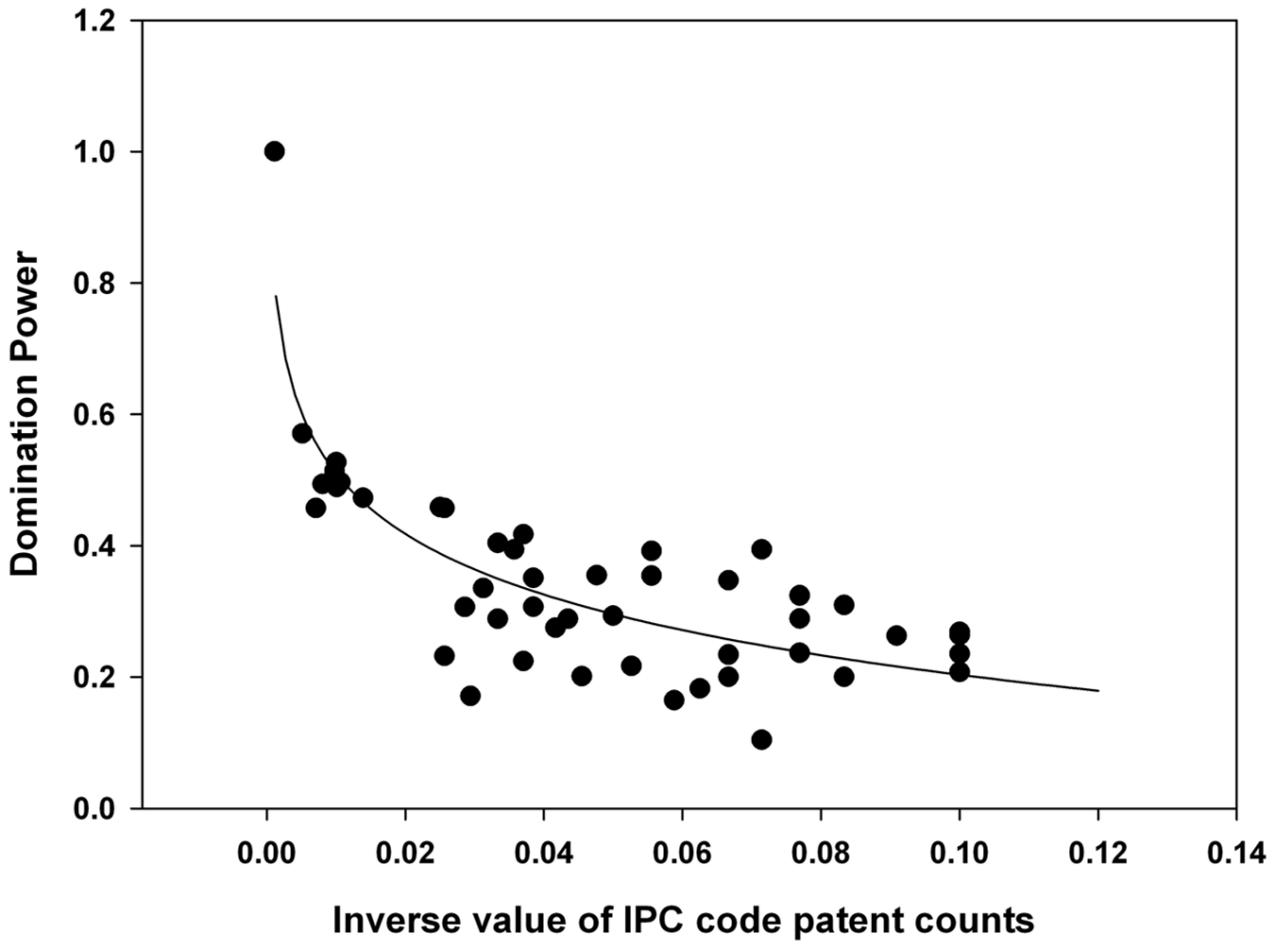
Nonlinear regression from 1976 to 2011 (fourth period), γ = 0.1332.

**Figure 15:**
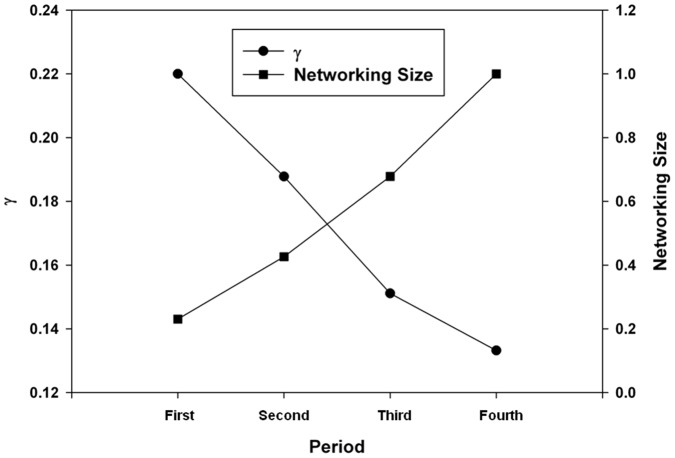
γ value variation and networking size.

To investigate the degree of technological convergence, nonlinear regression analysis was performed. The results of the analysis are depicted in Figures 11–15. Additionally, the statistical test of nonlinear regression is shown in Table 2. Most of the large nodes tend to have high Shannon entropy and binding force values, and vice versa. These graphs show that the γ value has decreased through the time periods. Over the total period, the magnitude of the networking size has also been maximized.

**Table 2:** The statistical test of nonlinear regression.

First period					
	Coefficient	Std. Error	Rsqr	t	P
c	0.2512	0.0867	0.6915	−2.8971	0.0081
γ	0.2200	0.0306	0.6915	−7.1801	<0.0001
Normality Test (Shapiro-Wilk)			Constant Variance Test		
Passed (P = 0.9988)			Passed (P = 0.9516)		

Second period					
	Coefficient	Std. Error	Rsqr	t	P
c	0.2215	0.0692	0.6383	−3.1995	0.0028
γ	0.1878	0.0229	0.6383	−8.1893	<0.0001
Normality Test (Shapiro-Wilk)			Constant Variance Test		
Passed (P = 0.6293)			Passed (P = 0.0535)		

Third period					
	Coefficient	Std. Error	Rsqr	t	P
c	0.1405	0.0523	0.6724	−2.6870	0.0101
γ	0.1511	0.0159	0.6724	−9.5021	<0.0001
Normality Test (Shapiro-Wilk)			Constant Variance Test		
Passed (P = 0.4949)			Passed (P = 0.5399)		

Fourth period					
	Coefficient	Std. Error	Rsqr	t	P
c	0.1033	0.0432	0.7039	−2.3902	0.0209
γ	0.1332	0.0126	0.7039	−10.5707	<0.0001
Normality Test (Shapiro-Wilk)			Constant Variance Test		
Passed (P = 0.8641)			Passed (P = 0.7108)		

The first period shows the highest γ value, and the magnitude of the networking size is smallest among all the periods (Figure 15). Therefore, the γ value and the magnitude of the networking size in the first period manifest their insufficient levels for technological convergence. Throughout all the periods, γ has decreased from 0.2200 to 0.1332. Furthermore, the magnitude of the networking size has been maximized. Thus, we can conclude that the disparity of interactions among various technology fields decreases and the overall networking size increases as technological convergence accelerates. Therefore, technological convergence is a type of co-evolutionary process that decreases the disparity among technology fields and simultaneously increases the technological interaction networks.

## Discussion and Conclusions

This study has investigated technological convergence in the case of patent citations in printed electronics by developing and applying the physical idea and concepts of entropy and gravity. We believe that the quadrants, consisting of the Shannon entropy and the binding force, indicate the unique patterns and trends of technological convergence. Furthermore, we have found idiosyncratic evolutionary patterns of technological convergence, consequently providing valuable insights and a blueprint for future technological developments.

Printed electronics is socially and industrially one of the representatives of technological convergence, which comprises various element technologies such as *substrate*, *circuit*, *ink*, *device*, and *control*. According to given time periods, the technology fields have been actively correlated and have raised their forces of attraction to one another. These technologies have also played an important role in technological convergence. In detail, a small number of *ink*-related technologies have demonstrated high Shannon entropy and binding force values, and they have been the leading core technologies for convergence. The *control*-related technologies have also indicated a propensity to link other technologies for convergence themselves. For this reason, we can assume that *control* technology has a helping characteristic. The *circuit*- and *substrate*-related technologies have opted to provide the basic element technologies to the other ones. The *device*-related technology possesses the largest number of patents; accordingly, it has the largest nodes among all the technology fields. Consequently, the *device* technology constructs and materializes technological convergence.

The analysis in this study offers insights and valuable implications for technological convergence and related technological development. We have found idiosyncratic, dynamic patterns of interactions among component technologies, based on the results of our analysis and considering the characteristics of the printed electronics industry. First, the active interactions among the technology fields constitute a necessary process for technological convergence. Second, in technological convergence, some technologies mediate among and integrate other technologies. Third, the basic technology field provides the platform for the technological development of convergence.

In summary, in the technological convergence process, heterogeneous technology fields are vigorously involved and maintain diverse interactions with one another, consequently being converged. In this process, the gap and disparity of the degree of interaction and attraction among the technologies are shown to decrease. This finding is evident from our nonlinear regression analysis; the γ value has decreased from the first period through the fourth period, which reveals the decline in the gap and disparity among the technologies. Furthermore, the magnitude of the networking size has been maximized. These results show that the printed electronics technology has evolved and advanced, with decreasing disparity among the component technologies. Moreover, the changing patterns of indexes (developed in our study to investigate the role of and interactions among technology fields) show the characteristic of converging dynamics. For this reason, printed electronics can be considered as a good model to explain technological convergence from the perspectives of both industry and technology. Based on our study's results, we have found unique patterns of technological convergence and the dynamics of the technological development of interactions among disparate technology fields. Indeed, we have discovered that technological convergence has the characteristics of a co-evolutionary process, which decreases the disparity among technology fields while increasing the technological interaction networks.

We expect this study to help the readers identify and understand the trends and patterns of technological convergence, based on our results. In this context, our study has implications for technology analysis, strategic technology management, and technology policy. First, we believe that our entropy and gravity concepts are applicable to other industries or technology areas. For example, from the initial cellular/feature phone to the present smartphone, the mobile phone-related technologies have a substantial history of technological developments. Therefore, the characteristics and roles of the component technologies of this industry can be studied using the entropy and gravity concepts. Moreover, the 3D printer industry and related technologies have attracted worldwide attention. At this stage, the component technologies of the 3D printer industry can be investigated from the perspective of technological convergence. It is also a possible area where we can apply our analytical indexes and network-specific approach to investigate the technological dynamics of domination power and technological disparity.

Second, from the results, we can draw some strategic implications for corporate technology management. In other words, this study suggests significant insights into the process of building a technology-strategic portfolio during technological convergence at the business level. The observed technological disequilibrium reveals that convergence occurs at a level below the maximum capacity of each component technology. Therefore, knowing the respective roles of the technology fields involved in convergence and understanding the changes of disparity among them are vital to establish corporate technology strategies.

Third, this study enables policymakers to determine the industry policy, considering the pace of technological development. In this regard, it is vital to investigate the technologies and the corresponding technology fields and areas central to the convergence process. These characteristics can be observed in printed electronics, and we identified the typical roles of and interactions among technology fields, which lead to technological convergence.

The fact that the position of the core technology changes during technological convergence suggests the need to establish the research and development strategy and policy from a dynamic perspective that is responsive to the development phase of technological convergence. Restated, a firm strategy and technology policy on technological convergence should be developed from the different perspectives from those associated with a more homogeneous technology development.
